# The comprehensive progress of tooth regeneration from the tooth development to tissue engineering and clinical application

**DOI:** 10.1186/s13619-025-00249-7

**Published:** 2025-07-31

**Authors:** Yi Sui, Ziqi Zhou, Siqi Zhang, Zhigang Cai

**Affiliations:** 1 Department of Oral Emergency and Oral Maxillofacial Surgery, Peking University School and Hospital of Stomatology, Haidian District, No.22, Zhongguancun South Avenue, Beijing, 100081 People’s Republic of China; 2 National Center for Stomatology, Beijing, People’s Republic of China; 3 National Clinical Research Center for Oral Diseases, Beijing, People’s Republic of China; 4 National Engineering Research Center of Oral Biomaterials and Digital Medical Devices, Beijing, People’s Republic of China; 5 Department of Central Laboratory, Peking University School and Hospital of Stomatology, Beijing, People’s Republic of China

**Keywords:** Tooth regeneration, Tissue engineering, Stem cells, Scaffold, Pulp regeneration

## Abstract

The advancement of tooth regeneration has offered revolutionary progress in the treatment of tooth defects and tooth loss, particularly in whole-tooth regeneration, pulp-dentin regeneration, and enamel regeneration. This review comprehensively analyzes the latest research progress in the biological foundations of tooth regeneration, stem cell applications, and tissue engineering technologies while discussing the prospects for clinical translation of these technologies. At present, pulp-dentin regeneration technology has entered clinical trials and demonstrated preliminary efficacy; however, the maturity and controllability of this technology require further enhancement. In situ whole-tooth regeneration has been achieved in animal models but still confronts ethical and functional challenges. Although the development of new materials has provided novel strategies for the epitaxial growth of enamel, enamel regeneration remains in its early stages. Tissue engineering technologies offer new avenues for tooth regeneration but still need to address issues such as immune rejection and long-term stability to realize the clinical application of tooth regeneration technologies.

## Background

Teeth are extremely valuable organs in the human body, performing essential functions such as chewing, speech, and supporting the soft tissues of the face, thereby maintaining facial form and appearance. Given their limited self-repair capabilities, teeth often struggle to heal themselves after trauma or inflammation. Tooth loss due to disease, trauma, or aging can severely affect the quality of life and overall well-being. Currently, the treatment for missing teeth primarily relies on restorative methods such as dental implants, dentures, or dental bridges. Although these methods can restore some functionality, they cannot fully replicate the biomechanical characteristics and sensory functions of natural teeth and carry certain risks of failure and complications (Ferreira et al. 2007). Therefore, exploring more effective tooth restoration methods from new perspectives holds significant clinical importance.

Tooth regeneration research aims to restore damaged dental tissue structures through biological means using stem cells (SCs) and tissue engineering technologies. In previous studies, we induced the induced pluripotent stem cells (iPSCs) with continuous stimulation of bone morphogenetic protein 4 (BMP4) and regenerated the entire dental tissue under the renal capsule of mice (Li et al. 2019). Wu et al. also successfully regenerated whole teeth in situ in a large animal model, a pig, by reassociating dental epithelial and mesenchymal cells (Wu et al. 2019). In addition to whole tooth regeneration, the regeneration of different dental tissues is also being investigated. In recent years, researchers have induced iPSCs to generate ameloblast-like cells and human ameloblast organoids, which demonstrated the potential for tooth regeneration when interacting with mesenchymal cells, offering new perspectives and methods for enamel repair (Alghadeer et al. 2023; Kim et al. 2023; Liu et al. 2016). The regeneration of the pulp-dentin complex is relatively mature in the laboratory phase and is advancing to the clinical stage (Kobayashi et al. 2022; Zhang et al. 2020a). To date, over 20 studies have reported related clinical trials, achieving pulp regeneration containing the odontoblast layer, blood vessels, and nerves in teeth, formation of new dentin, and preservation of sensation to thermal and electric stimuli (Nakashima and Iohara 2017; Xuan et al. 2018). Other dental tissue regeneration still faces bottleneck issues before entering the clinical stage. The regeneration of cementum is often associated with the regeneration of periodontal tissues. Because the development of periodontal tissues differs from tooth development and has its distinct systemic characteristics, the subsequent parts of this review will not expand on this issue.

**Fig. 1:**
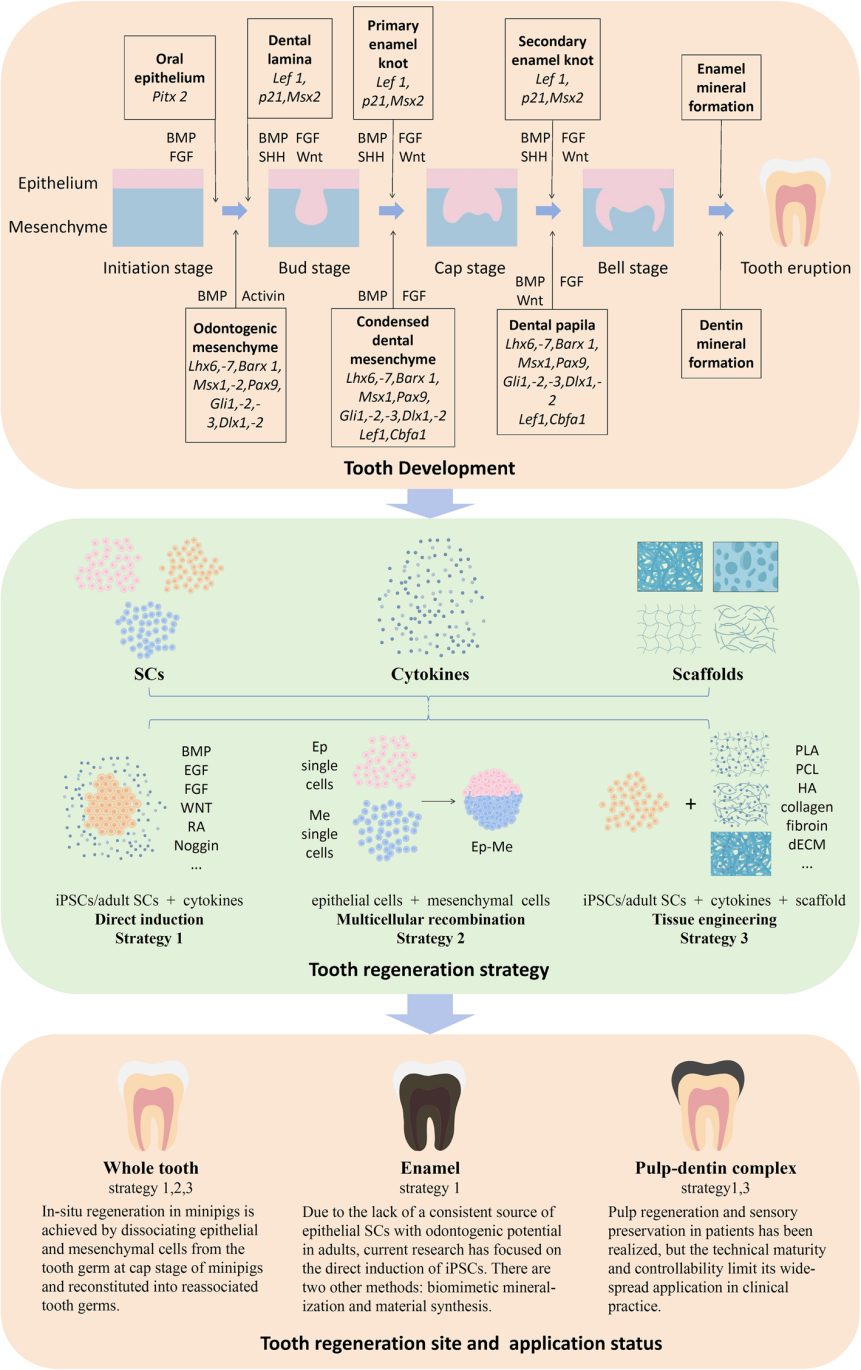
Schematic of tooth regeneration process

Briefly, tooth regeneration research holds promise in achieving in situ tooth regeneration, including whole tooth regeneration, pulp-dentin regeneration, and enamel regeneration, providing safer, more effective, and longer-lasting treatment options for missing teeth, damaged teeth, and related diseases. Scientists are employing stem cells, cytokines or/and materials by three main strategies to achieve tooth regeneration (Zhang and Yelick 2021). The first strategy, Direct Induction Strategy, employs a suite of signaling molecules to facilitate the differentiation of stem cells into specific cell types integral to dental structures, thereby promoting regeneration. The second one, termed the Multicellular Recombination Strategy, involves the synergistic combination of various stem cells with signaling molecules to emulate the intricate interactions that occur during natural tooth development, enhancing the regenerative process. Lastly, the Tissue Engineering Strategy encompasses the intricate integration of stem cells, biomaterials, and cytokines to construct a three-dimensional scaffold designed to support and enhance tooth regeneration, with the ultimate goal of achieving comprehensive restoration of both hard and soft tissues. Therefore this review bases on tooth development, summarizes disassembly the application of three key elements, stem cells, cytokines and materials, in the tooth regeneration, and focuses on the clinical translation of tooth regeneration to reveal the research status and development prospects of tooth regeneration (Fig. 1).

## Biological basis of tooth regeneration

Tooth development is a series of interactions between the ectodermal epithelium and the mesenchyme (Zhang et al. 2005). The continuous interactions that control tooth development and patterning are highly complex and not yet fully elucidated. Various signaling molecules, including inductive and morphogenetic factors, are expressed during tooth development. The research indicates that signaling molecules such as Wnt, BMP, fibroblast growth factor (FGF), and sonic hedgehog (SHH) play significant roles in this process (Zhang et al. 2023; Zhang et al. 2005). They interact with each other and are sequentially expressed spatially and temporally to regulate tooth morphogenesis and maturation of dental tissues (Alghadeer et al. 2023; Balic 2019; Balic and Thesleff 2015; Lan et al. 2014).

In the early stages of tooth development, the epithelial and mesenchymal cells have not yet made contact. At this time, the expression of key transcription factors such as Pitx2, Lef1, and Pax9 commenced, laying the foundation for subsequent tooth formation (Balic 2019). These factors take on an important role in determining the region of tooth development, activating the expression of related genes to specify the location of tooth development (Yu et al. 2020). Signaling pathways such as BMP and FGF are involved in the positioning of tooth development by regulating the expression of these factors, for example, the expression of Pax9 is induced by Fgf8 and inhibited by BMP2 and BMP4 (Neubuser et al. 1997).

The initiation of tooth development requires interactions between the epithelial and mesenchymal cells (Zhang et al. 2005). When the dental epithelium (DE) derived from the oral epithelium (OE) contacts the mesenchyme derived from the neural crest, a series of signaling pathways are activated, including Wnt, BMP, FGF, and SHH. Wnt signaling activates downstream genes by stabilizing β-catenin, promoting tooth induction and early morphogenesis (Liu et al. 2008a). BMP signaling regulates tooth morphogenesis by affecting the differentiation and proliferation of mesenchymal cells (Liu et al. 2022). Alghadeer et al. discovered that BMP signaling plays a key role in the transition from the OE to the DE and from the DE to the outer enamel epithelium (Alghadeer et al. 2023). FGF signaling is involved in epithelial cell proliferation and tooth bud formation (Huang et al. 2019). SHH influences multiple cellular behaviors during tooth development, while Gli family, as its downstream molecule, responds to SHH signaling and regulates relevant gene expression (Hosoya et al. 2020).

As tooth development progresses, epithelial cells form the enamel organ, and mesenchymal cells differentiate into the dental papilla. At this time, the formation of the enamel knot is crucial for shaping the tooth (Zhang et al. 2023). The enamel knot is a signaling center in tooth development, which regulates tooth shape and size by secreting signaling molecules such as FGF4, BMP4, Wnt10B, Shh, and p21 and transcription factors such as Msx1. Key transcription factors such as Msx1, Barx-1, and Dlx1 play a central role in tooth morphogenesis, determining tooth type through interactions with signaling pathways such as BMP and FGF (Neubuser et al. 1997; Tucker et al. 1998).

The subsequent stages involves dentin formation and enamel mineralization. At this stage, the BMP signaling pathway plays a key role in odontoblast differentiation. BMPs promote the proliferation and differentiation of postnatal human dental pulp SCs (DPSCs) into odontoblasts and facilitate the synthesis and secretion of the dentin matrix. Conversely, they regulate the expression of dentin-related genes such as dentin sialophosphoprotein (*Dspp*) and dentin matrix protein 1 (*Dmp1*) by controlling the expression of transcription factors such as distal-less 3 (Dlx3), Osx, and Runx2 (Liu et al. 2022). During the enamel mineralization stage, the Wnt signaling pathway promotes enamel mineralization by, affecting the expression of specific genes such as the SP6 transcription factor, which subsequently affects the expression of enamel proteins such as AMBN and amelogenin (AMELX) (Alghadeer et al. 2023).

DNA methylation and histone modification play regulatory roles at various stages of tooth development (Chen et al. 2023). These epigenetic markers influence gene expression, thereby finely regulating cell fate determination and differentiation during tooth development. For example, Jing et al. (2019) found that Ezh2 regulated gene expression by methylating histone H3 at lysine 27 (H3K27me3), thereby affecting tooth shape and number. Zeng et al. (2022) proved that non coding RNAs, such as microRNAs and long non-coding RNAs, are also involved in the regulation of various aspects of tooth development, affecting mRNA stability and translation to regulate gene expression.

Recent studies have shown that post—translational modifications, such as ubiquitination, play a crucial role in tooth development. For instance, Smurf1, an E3 ubiquitin ligase, influences tooth development by ubiquitinating and degrading active RhoA to regulate ameloblast polarity. Additionally, ubiquitination is involved in ameloblast apoptosis regulation, which is essential for normal tooth development (Niu et al. 2022). The E3 ubiquitin ligase, murine double minute 2 promotes the odontoblast-like differentiation of DPSCs by mediating ubiquitination of the transcription factor Dlx3, which is essential for Dlx3 transcriptional activity on *Dspp* as well as subsequent odontoblast-like differentiation and dentin formation, and ubiquitinating p53, which eliminated the inhibitory effect of p53 on the odontoblast-like differentiation of DPSCs (Zheng et al. 2022; Zheng et al. 2020). While, WW domain-containing E3 Ubiquitin-protein ligase 2 promotes odontoblast differentiation by targeting phosphatase and tensin homolog (PTEN) for degradation, which relieves PTEN's inhibition of Kruppel-like factor 5 (KLF5) transcriptional activity on dentin matrix genes *Dspp* and *Dmp1* (Fu et al. 2022, 2021). Delving into the mechanisms of epigenetic modifications and post—translational in tooth development can enhance our understanding of its molecular basis and offer new insights for tooth regeneration.

Signaling molecules in tooth development can maintain tissue homeostasis and regulate SCs fate. Thus, understanding the molecular mechanisms of tooth development provides a theoretical basis for inducing the differentiation of SCs into tooth-related cells. Some studies have induced iPSCs differentiation by simulating the expression of key signaling pathways and transcription factors during tooth development to achieve tooth regeneration (Li et al. 2019).

## SCs for tooth regeneration

### iPSCs

iPSCs, derived from somatic cells through reprogramming with transcription factors, possess characteristics similar to embryonic SCs and can differentiate into various cells (Takahashi and Yamanaka 2006), including those required for tooth development, such as epithelial and mesenchymal cells. This has opened up broad application prospects in tooth regeneration (Otsu et al. 2014) (Table 1).

**Table 1:** Application of iPSCs in tooth regeneration

Sources of the IPSC	Induction strategy	Model	Regenerated result		Ref
			cell	tissue	
mouse iPSCs line	ASF-BMP4 → ASF-CM	in vitro, mouse	ameloblast and odontoblast cells^*^	epithelium and odontoblast T^**^	Liu et al. 2016
	iNCLCs → transfected Pax9/Bmp4 expression plasmids	in vitro	odontoblast cells		Seki et al. 2015
	+ DE SF2-24 cells	in vitro	Ambn-expressing dental epithelial cells		Arakaki et al. 2012
mouse gingiva-derived iPSCs	iNCLCs → + human tooth scaffolds	in vitro, mouse	odontoblast cells	dental pulp T, V	Zhang et al. 2020a
mouse gingival fibroblast	EBs → + BMP4 + RA + LiCl → + TGF-β1 + EGF + LiCl	in vitro	ameloblast cells		Miao et al. 2021b
mouse embryonic fibroblast	iNCLCs → + BMP4 + FGF8 + Wnt3a	in vitro	odontoblast cells		Takada et al. 2022
hdDPSCs	MMC treated HERS-SV40 layer	in vitro	dental epithelial SCs		Kim et al. 2020
hDPSCs	EBs → + BMP4 → + PLLA scaffolds/extract of bioactive cement	in vitro, mouse	odontoblast cells	pulp T	Xie et al. 2018
human urine cells	RA + BMP4 → + mouse dental mesenchyme	mouse		tooth-shaped structures	Cai et al. 2013
human dermal fibroblasts or gingival fibroblasts	CNCLCs → selection and passage → osteo/odontogenic differentiate + FGF4&FGF9 → + human root fragment + SLan	in vitro, mouse	odontoblast cells	dental pulp T, V	Kobayashi et al. 2022
human skin fibroblast	BMP4 → GSK-I + EGF + LDN + NT4 → GSK-I + EGF + BMP4 + TGF-β1 → + DPSCs	in vitro, mouse	early ameloblast organoids		Alghadeer et al. 2023
human cord blood	EB → BMP4 + RA → Noggin + EGF → BMP4 + EGF → + dental mesenchyme	in vitro, mouse	dental epithelial cell		Kim et al. 2021
	EB → BMP4 + RA → + Noggin + EGF → BMP4 + EGF; EB → B27 + insulin + bFGF + EGF → + GelMA, collagen, and agar gel	in vitro, mouse		cartilage and bone T, bioengineered tooth-shaped structure	Kim et al. 2024
	EBs → RA + BMP4 → Noggin + EGF → Organoid culture → –Noggin + BMP4	in vitro, mouse		human ameloblast organoid	Kim et al. 2023

*T* tissue, *V* blood vessels

^*^in vitro; ^**^in vivo

#### Applications

In recent years, scientists have capitalized on iPSCs pluripotency to differentiate them into dentin-like tissue, pulp-like tissue, and periodontal tissue under specific induction factors and culture conditions, demonstrating great potential in tooth tissue regeneration. Cai et al. successfully generated tooth-like structures from integration-free human urine-iPSCs, which possessed normal structures, including ameloblasts and enamel derived from the epithelial component, and dentin, pulp, and cementum developed from the mesenchymal component (Cai et al. 2013). The enamel is the hardest tissue in the human body, and once damaged, it cannot self-repair because of its highly mineralized characteristics. The lack of a consistent source of epithelial SCs with odontogenic potential in adults limits enamel regeneration, and the iPSCs research has introduced new possibilities for the regeneration of this critical dental component. Researchers have successfully differentiated iPSCs into ameloblast-like cells through coculture with immortalized dental epithelial cell lines (IDECL), application of the conditioned medium from IDECL, replication of the sequential steps through key developmental mechanisms, as well as coculture with dental mesenchymal cells (Hermans et al. 2024; Kim et al. 2020). They have generated organoids capable of secreting enamel proteins in vitro (Alghadeer et al. 2023), which is a crucial step for tooth repair and regeneration. Moreover, iPSCs possess the plasticity to differentiate into cranial neural crest-like cells (CNCLCs), which give rise to various dental cell types, including odontoblasts and periodontal ligament (PDL) cells. The differentiation of iPSCs into CNCLCs and their subsequent maturation into functional odontoblasts has been confirmed in vitro, leading to the formation of organized dental pulp-like structures. These structures exhibit characteristics reminiscent of native dental pulp, including the presence of odontoblasts, vascularization, and production of extracellular matrix (ECM) components (Kobayashi et al. 2022). The potential of iPSCs in periodontal regeneration is also noteworthy. iPSCs can be directed to differentiate into PDL-derived mesenchymal stromal cell-like cells, which are crucial for the regeneration and repair of periodontal tissues (Wang et al. 2024). Recently, Kim et al. conducted a series of experiments and successfully differentiated hiPSCs into dental epithelial cells and dental mesenchymal cells. By utilizing biomaterials such as GelMA, collagen, and agar gel, they facilitated the mineralization of these cells to form hard tissues resembling teeth and bone. Moreover, the regenerated tissues were shown to integrate well with the surrounding tissues after in vivo transplantation. This study provides novel techniques and approaches for utilizing hiPSCs in dental and bone regeneration, offering valuable insights for future clinical regenerative medicine (Kim et al. 2024).

Considering that the source difference of iPSCs may introduce genetic and epigenetic alterations affecting their differentiation potential (Liu et al. 2016), numerous studies have reprogramed cell populations from various dental tissues into iPSCs (Hynes et al. 2015). For instance, Yan et al. successfully reprogramed iPSCs from SCs from human exfoliated deciduous teeth (SHED), apical papilla (SCAPs), and DPSCs (Yan et al. 2010). Klymkowsky et al. (2010) obtained iPSCs from human gingival fibroblasts and found that the reprograming efficiency of fibroblasts from easily accessible gingival tissue was more than seven times higher than that of fibroblasts from the tail tip. Li et al. discovered that human gingival fibroblast-derived iPSCs exhibited a greater tendency toward periodontal differentiation than human neonatal skin fibroblast-derived iPSCs (Li et al. 2018). These studies further demonstrate the unique advantages of dental tissue-derived cells in oral tissue regeneration and their potential as a cell source for iPSCs for examining the fundamentals of cell reprograming and future clinical applications.

#### Limitations

Despite the promising potential of iPSCs in tooth regeneration, several challenges must be addressed to harness their full potential. First, the differentiation process of iPSCs requires precise control. Teeth are composed of different cells, including odontoblasts, cementoblasts, pulp cells, and ameloblasts. Each cell type possesses distinct functional and morphological characteristics, necessitating the ability of iPSCs to differentiate into these specific cell lineages. A complex network of signaling pathways regulates this process. The precise temporal and spatial activation and inhibition of these signaling pathways are crucial for guiding iPSCs differentiation (Yoshizaki et al. 2020). In addition, culture conditions such as oxygen concentration, substrate stiffness, cell density, and medium composition can affect the efficiency and maturation of iPSCs differentiation. Second, iPSCs may undergo mutations during culture, which could affect their differentiation capacity and the stability of regenerated tissues, leading to heterogeneity and unpredictability in iPSCs applications. Third, although autologous iPSCs theoretically avoid immune rejection, changes in cell surface antigen expression during culture and differentiation could elicit immune responses, even when using a patient’s cells to generate iPSCs. Furthermore, the use of oncogenic factors for reprograming endows iPSCs with the ability to differentiate into various cell types, which also implies a risk of tumorigenesis (Bulcha et al. 2021). Therefore, rigorous quality control measures and safety assessments are necessary to ensure the therapeutic safety of iPSCs-derived dental cells. The use of nonintegrating vectors can mitigate some of these risks (Bulcha et al. 2021). Currently, the long-term stability and functionality of regenerated tissues remain unresolved issues. The regenerated tissues must integrate with existing alveolar bone and periodontal tissues and withstand masticatory forces. However, whether iPSCs-derived cells can fully mimic the morphology and function of natural odontogenic cells and whether they can achieve sufficient maturity to function in regenerated tissues are unclear. Thus, future studies are needed to further explore these challenges and promote the clinical translation of iPSCs applications in tooth regeneration.

### Adult SCs

Adult SCs with low risk have been used as an alternative to regenerative medicine in the intestine (Qi et al. 2020), pancreas (Kou et al. 2022), heart (Sadek and Olson 2020), bone (Iaquinta et al. 2019), and lung (Chen et al. 2021b). In tooth regeneration, research and application of adult SCs are becoming a key force in driving the regeneration of dental pulp, dentin, enamel, and periodontal tissues. This section provides a comprehensive analysis of the application and progress of adult SCs from different sources in the regeneration of different tooth layers (Table 2).

**Table 2:** Application of Adult SCs in tooth regeneration

Cell types	Study design	Model	Regeneration results	Ref
DPSCs	DPSCs + USPIO labeled silk fibroin/HA scaffold implanted into dental fragments	mice	dentin pulp T, V	Zhang et al. 2019
	3D DPSC constructs inserted into treated root canal	mice	dental pulp T, V	Itoh et al. 2018
	vascularized DPSC constructs(endothelial differentiated cell constructs) placed in treated root canal	mice	dental pulp T, V	Katata et al. 2021
	DPSC aggregate implanted into young permanent incisors	mice, minipigs, human	dental pulp T, V, N	Xuan et al. 2018
SHED	SHED aggregates implanted into porcine permanent incisors	minipigs	dental pulp T, V, N	Guo et al. 2020
	HIF-1α-stabilized SHED + PuraMatrix hydrogel injected into the root canals of human tooth fragments	mice	dental pulp T, V	Han et al. 2022
SCAP,PDLSC	HA/TCP block containing SCAP + Gelfoam containing PDLSCs inserted into the socket	minipigs	bioroot	Csete et al. 2006
SCAP,DPSC	SCAP/DPSC + PLG scaffolds inserted into the canal space of each root fragment	mice	dental pulp T	Huang et al. 2010
DFC	DFSCs + PCL scaffold transplanted into the defects	rat	bone T	Rezai-Rad et al. 2015

*T* tissue, *V* blood vessels, *N* nerves

DPSCs, extracted from the pulp of adult teeth, are known for their high proliferative capacity and multilineage differentiation potential (Gronthos et al. 2000). They are currently the most widely used adult SCs in tooth regeneration. Under the induction of specific biomaterials and cytokines, DPSCs can differentiate into neural cells, adipocytes, vascular endothelial cells, etc. (Bar et al. 2021). They can also differentiate into odontoblast-like cells and form mineralized hard tissues, providing a potential approach for the repair of the pulp-dentin complex (Itoh et al. 2018; Katata et al. 2021; Zhang and Yelick 2021; Zhang et al. 2019). Consequently, DPSCs are the most frequently utilized in the regeneration research of the pulp-dentin complex and have been applied in clinical trials (Meza et al. 2019; Nakashima et al. 2017; Nakashima and Tanaka 2024). DPSCs have low immunogenicity (Tatullo et al. 2015), ease of accessibility (Wang et al. 2010), and high differentiation potential; thus, they are also employed in periodontal regeneration (Ferrarotti et al. 2018). SHEDs are extracted from the pulp of exfoliated primary teeth in children. Similar to DPSCs, SHEDs can form tissue resembling the pulp-dentin complex and exhibit higher proliferative activity and stronger osteogenic potential (Guo et al. 2020; Han et al. 2022; Miura et al. 2003; Nakamura et al. 2009; Suchánek et al. 2010). They have also been utilized in clinical trials for the regeneration of the pulp-dentin complex (Xuan et al. 2018). For instance, Guo et al. implanted aggregates of human dental pulp stems cells (hDPSCs) from deciduous teeth with decellularized tooth matrix into the dislocated teeth to form bioengineered teeth and replanted them, showing successful regeneration of functional pulp and periodontal tissue, and no adverse effects were observed during the 24-month follow-up (Guo et al. 2021). In addition, clinical trials using human DP mesenchymal SCs for the treatment of chronic periodontitis have received implicit approval, signifying the formal entry of human dental pulp mesenchymal SCs as a therapeutic agent into the clinical exploration phase.

Various adult SCs, such as SCAPs, dental follicle cells (DFCs), and PDL SCs (PDLSCs), have also been used in tooth regeneration. SCAPs are SCs located in the apical papilla of immature permanent teeth, whereas DFCs originate from developing dental follicles. Both are cells derived from developmental tissues and may exhibit greater plasticity than other SCs (Kang et al. 2019; Volponi et al. 2010). The former has shown significant potential in pulp regeneration, whereas the latter has demonstrated its ability to regenerate complete periodontal tissues (Guo et al. 2013; Huang et al. 2010; Rezai-Rad et al. 2015). PDLSCs, extracted from the PDL, have been utilized for the reconstruction of the periodontal complex, including the alveolar bone, new cementum, and PDL (Iwasaki et al. 2018; Liu et al. 2008b; Seo et al. 2004; Tian et al. 2021; Zhao et al. 2020). Sonoyama et al. successfully generated a root-periodontal complex that can support the tooth crown by coculturing SCAPs (for root formation) and PDLSCs (for periodontal tissue formation) and transplanted it into a mini pig model (Csete et al. 2006). In addition, bone marrow mesenchymal SCs (BMSCs), adipose-derived SCs (ADSCs) and gingival mesenchymal SCs (GMSCs) have been applied in tooth regeneration research, and the nature and potential uses of these cells still require further investigations (Balaban et al. 2024; Hung et al. 2011; Luo et al. 2023).

In other words, adult SCs show great potential in tooth regeneration. DPSCs, SHED, and SCAPs have made significant progress in dental hard tissue and pulp regeneration; PDLSCs show application prospects in periodontal tissue regeneration; DFCs show good effects on both periodontal and bone regeneration. The research and application of DPSCs and SHED are rapidly developing and gradually transitioning toward clinical translation, providing new strategies and hope for dental treatments, with the potential to become an indispensable part of dental therapy in the future. However, teeth are complex three-dimensional structures, including hard tissues (such as dentin and enamel) and soft tissues (such as pulp and PDL). Direct use of SCs for induced regeneration to form fine structures is challenging. Tissue engineering technology (precise cytokines, multicellular recombination and biomaterials) was introduced to simulate this complex structure.

## Cytokines

In tooth regeneration, the addition of cytokines plays a critical role in guiding cell differentiation and tissue formation.

In direct induction strategy, cytokines are typically introduced in temporally sequenced combinations to achieve specific regenerative objectives. Research demonstrates that distinct cytokines exert stage-specific effects that mimic natural developmental processes and enhance regeneration outcomes. BMP4 significantly promotes dentinogenic and amelogenic cell formation during early stages, while retinoic acid (RA) facilitates odontogenic epithelial cell development by modulating neural ectodermal cell differentiation pathways (Liu et al. 2016; Metallo et al. 2008). Furthermore, the combined application of FGF8 and Wnt3a significantly enhanced the differentiation of adult dentin cell-like tissues at a later stage (Takada et al. 2022).This temporal control ensures optimal factor combinations at each stage and maintains normal cellular differentiation by regulating the activation and suppression of signaling pathways. For instance, Miao et al. established a three-phase protocol for differentiating iPSCs into ameloblasts. The initial stage employed Nodal signaling inhibition via SB431542 combined with BMP4 to induce surface ectoderm formation. Subsequent phases utilized RA, BMP4, and LiCl (a Wnt/β-catenin signaling pathway activator) to promote odontogenic epithelial specification, followed by moderated Wnt inhibition (e.g., by decreasing LiCl concentration) alongside EGF and TGF-β1 to drive terminal ameloblast differentiation (Miao et al., 2021a). This temporal control ensures the optimal combination of factors at each stage, maintains normal cell differentiation by precisely regulating the activation and shutdown of signaling pathways, and regulates the composition and structure of the extracellular matrix, as well as cell–cell interactions, to provide a suitable microenvironment for cell differentiation. This precise timing control not only improves differentiation efficiency, but also enhances the specificity of the regenerating tissue, ultimately achieving efficient tooth regeneration. In this way, researchers were able to gradually guide the cells from epidermal ectoderm to odontogenic epithelial cells, and ultimately differentiate them into functional enamel-forming and dentin-forming cells, providing a powerful tool for tooth regeneration.

In multicellular recombination strategy, the addition of cytokines is typically limited or absent, with tooth-specific cell development and maturation primarily driven by cell–cell interactions. Notable examples include the reconstitution of epithelial sheets generated by integration-free human urine iPSCs with mouse dental pulp mesenchymal tissue, where direct cellular communication induced tooth-like structures without exogenous factors (Cai et al. 2013). This intercellular synergy not only reduced the use of exogenous factors, but also improved the stability and functionality of the regenerated tissue.

In tissue engineering strategy, cytokines are primarily added to achieve specific regenerative goals, such as promoting cell proliferation, differentiation, and homing. Some studies have loaded cytokines onto scaffolds, as exemplified by collagen gel loaded with BMP7 that significantly promoted stem cell migration, odontogenic differentiation, and angiogenesis, and successfully induced hDPSCs to form vascularized pulp-like tissue (Liang et al. 2022a). And in 3D bioprinting technology, some complex bioinks may integrate cytokines, including BMP-mimetic peptides tethering bioinks that maintain sustained bioactivity and promote dentin sialophosphoprotein (DSPP) and osteocalcin expression in printed constructs. These results suggest that BMP-mimetic peptides tethering bioinks can accelerate the differentiation of hDPSCs in 3D bioprinted tooth constructs (Park et al. 2020).

Cytokine-mediated cell homing strategies present clinically advantageous alternatives to cell transplantation. Cell homing is a more indirect method that relies on the attraction of biomaterials and bioactive molecules (such as BM7) to attract SCs in the body to the damaged or regenerative areas (Liang et al. 2022a). For cell homing strategies, the classic tissue engineering triad can be adjusted to resident SCs, customized scaffolds, and cytokines (Gallen and Widbillen 2017). Kim et al. developed microchanneled scaffolds delivering stromal-derived factor-1 (SDF1) and BMP7, demonstrating enhanced endogenous cell recruitment and vascularization (Kim et al. 2010).Compared with cell transplantation, this approach circumvents challenges associated with isolation of cells from the patient’s body or culturing them in vitro before transplantation. Consequently, it also avoids the difficulties associated with obtaining regulatory approvals. It facilitates the development of off-the-shelf products, is more cost-effective, and represents a superior alternative (Yildirim et al. 2011).

Additionally, exosomes, a critical paracrine medium that can carry bioactive molecules such as mRNAs, miRNAs, and proteins, which can be added as a specialized multi-component cytokine to modulate the functions of target cells in tooth regeneration (Cooper et al. 2020). Recent studies have discovered that DPSCs can facilitate tissue repair by replacing damaged cells and secreting paracrine factors. Dental pulp stem cell-derived exosomes (DPSC-exos), particularly those from osteogenic DPSCs (DPSC-OD-exos) were found to promote the odontogenic differentiation of DPSCs by transferring specific miRNAs and achieving the regeneration of pulp-like tissue in a semiorthotopic model (Hu et al. 2019; Huang et al. 2016). DPSC-exos can reduce immune reactions and stemness loss during in vitro manipulation compared to cellular therapies. Therefore, their integration with biomaterials may represent an effective and safe alternative strategy for tooth regeneration (Wang et al. 2020; Zhang et al. 2020b), though mechanistic details require further elucidation.

The application of cytokines varies significantly across regeneration strategies. In direct induction, a temporally sequenced combination of cytokines significantly improves the efficiency and specificity of cell differentiation; in multicellular recombination, cell–cell interactions are key to achieving tooth-specific cell development; and in tissue engineering, cytokine additions are mainly used to promote cell proliferation, differentiation, and homing, leading to efficient tooth regeneration. Future research should optimize cytokine combinations and application strategies to advance clinical translation of tooth regeneration technologies.

## Scaffolds

In addition to seed cells and cytokines, scaffolds also constitute an integral part of tooth regeneration research, collectively forming the cornerstone of tissue engineering studies. The scaffold provides structural support and an appropriate microenvironment for tooth regeneration, promoting sequential development and mineralization (Langer and Vacanti 1993) (Table 3).

**Table 3:** Application of Tissue engineering technology in tooth regeneration

Model	Study design				Regeneration effects	Ref
	Material	Technology	Stem cells	cytokines		
in vitro	Fibrinogen + gelatin + HA + glycerol	3D bioprinting	DPSCs		3D dental pulp T, enhance odontogenic differentiation	Han et al. 2019
	Alginate + dentin matrix	3D bioprinting	SCAPs		enhance odontogenic differentiation	Athirasala et al. 2018
	A fibrinogen-gelatin mixture + demineralized dentin matrix	3D bioprinting	DPSCs		3D human tooth-shaped construct, enhance odontogenic differentiation	Han et al. 2021
	DPSC-Exosomes		hDPSCs		promote odontogenic differentiation	Hu et al. 2019
mice	USPIO labeled silk fibroin/HA scaffold	Nanotechnology	DPSCs		dental pulp T, V	Zhang et al. 2019
	GelMA microfibers		DPSCs + HUVECs		dental pulp T, V	Liang et al. 2022b
	3D collagen fibrous porous scaffolds	Electrospinning	hDPSCs		dental pulp T, V	Zhang et al. 2022
	Porcine dental pulp dECM hydrogel		hDPSCs		dental pulp T, V	Yuan et al. 2023
	HPCH/CW/Exo hydrogel		hDPSCs		dental pulp T, V	Wang et al. 2023
	decellularized submandibular gland ECM		hDPSCs		dental pulp T, V	Shi et al. 2023
	OM-SHED derived extracellular vesicles-encapsulated hydrogel		hDPSCs		dental pulp T	Lu et al. 2024
	Porcine decellularized nerve matrix hydrogel		hDPSCs		dental pulp T, V, N	Liang et al. 2024
	Type I collagen hydrogel	Nanotechnology	miR@TDNs-treated DPSCs + HUVECs		dental pulp T, V	Wei et al. 2024
	Nanofibrous PLLA scaffold	Electrospinning	hDPSCs		dentin T	Wang et al. 2011
	GelMA microspheres	Electrostatic microdroplet technique	hDPSCs		dental pulp T, V	Yang et al. 2021
	DPSC-Exosomes		hDPSCs		dental pulp T, V	Huang et al. 2016
	Decellularized human tooth scaffolds		donor-matched Alx3-restored MSCs		dentin pulp T, V	He et al. 2019
	Fibrin gel		pDPSCs	PA-CM	dental pulp T	Choung et al. 2013
rat	Nanofibrous spongy microsphere/PLLA	Nanotechnology	hDPSCs		dental pulp T, V	Kuang et al. 2016
	Exosome-like vesicles		Dental papilla cells		dentin pulp T, V	Zhang et al. 2020b
	Human amniotic membrane ECM		hDPSCs		dental pulp T, V	Bakhtiar et al. 2022
	Cerium oxide nanoparticles/DMP1/Hydrogel	Surface modification techniques, Nanotechnology	hDPSCs		dentin	Zhao et al. 2024
	GelMA hydrogel	3D bioprinting	dental epithelial and dental mesenchymal cell		bioengineered tooth buds	Smith et al. 2018
	GelMA hydrogel	3D bioprinting	dental epithelial and dental mesenchymal cell, HUVECs		bioengineered tooth buds	Monteiro et al. 2016
dog	Silk fibroin scaffolds		DPSCs	SDF-1α	dental pulp T, V, N	Yang et al. 2015
	Collagen gel		dDPSCs	BMP7	dental pulp T, V	Liang et al. 2022a
	Collagen TE		pulp, bone marrow, adipose CD31- side population cells	SDF-1	dental pulp T, V, N	Ishizaka et al. 2012
	Atelocollagen		DPSCs	G-CSF	dental pulp T, V, N	Hara et al. 2013
	Atelocollagen	Nanotechnology	MDPSC	trypsin	dental pulp T, V, N	Iohara et al. 2020
	Collagen		CD105^+^ SCs	SDF-1	dental pulp T, V, N	Iohara et al. 2011
	Atelocollagen		DPSC/BMSC/ADSC	G-CSF	dental pulp T, V, N	Murakami et al. 2015
pig	Hyaluronic acid gel or collagen TE gel		sDPSCs		dental pulp T, V	Zhu et al. 2018
			SHED		dental pulp T, V, N	Xuan et al. 2018
	Decellularized tooth matrix		pDPSCs		functional teeth	Guo et al. 2021

*T* tissue, *V* blood vessels, *N* nerves

### Materials

The scaffold materials can be divided into natural and synthetic biomaterials. Different biomaterials possess distinct properties, and understanding these characteristics aids in selecting the most suitable biomaterial for the regeneration of the desired tissue, thereby enhancing the efficacy of tooth regeneration.

Natural biomaterials for scaffolds in tissue engineering are derived from natural sources, offering good biocompatibility and bioactivity that mimic the ECM environment, promoting cell adhesion, proliferation, and differentiation. These materials include polysaccharides, proteins and peptides, and ECM matrix derivatives. Decellularized ECM (dECM) scaffolds are a hot topic of current research. These materials are derived from natural tissues and have removed cellular structures, retaining the original tissue’s 3D structure and bioactive molecules, which are conducive to cell migration and tissue regeneration. Many preclinical studies have proven its regenerative potential in tissue repair; however, further studies are needed to refine the properties of the scaffold, assess its long-term safety, and promote its clinical application (Grawish et al. 2022; Zhang et al. 2017). For instance, research has prepared a decellularized matrix hydrogel derived from porcine dental pulps (pDDPM-G), which has shown superior performance in promoting odontogenesis, angiogenesis, and neurogenesis of the regenerating pulp-like tissue (Liang et al. 2024).

Synthetic biomaterials, prepared through chemical synthesis, offer strong plasticity, high stability, and batch production capabilities. Polymers such as polylactic acid (PLA), polycaprolactone (PCL), and polylactic-co-glycolide (PLGA) are highly utilized in tooth regeneration because of their customizability to meet specific size, shape, and mechanical property requirements and their excellent biocompatibility (Dhandayuthapani et al. 2011; He et al. 2016; Wang et al. 2017). Bioactive ceramics, such as HA and tricalcium phosphate, are widely used for bone defect repair because of their bone-inducing properties (de Grado et al. 2018). A study utilized 3D-printing technology to fabricate PCL/HA nanoparticles/diacrylate poly(ethylene glycol) scaffolds, which were subsequently evaluated in vitro using hDPSCs. The results indicated that these scaffolds possess significant potential for application in bone regeneration (Sousa et al. 2022).

Although natural biomaterials offer many advantages, they also have limitations, including batch-to-batch variability and challenges in processing and sterilization. Synthetic materials, despite their hydrophobic nature and limited bioactivity, can be tailored to specific applications and are more consistent in their properties. Each component has advantages and limitations. To overcome these challenges, researchers often combine natural and synthetic materials to obtain composite materials to improve their performance. For example, Chiu et al. studied the osteogenic properties of 3D scaffolds combined with mineral trioxide aggregate and polycaprolactone in tissue regeneration applications. Future research must further explore and optimize these materials to achieve more effective tooth tissue regeneration (Chiu et al. 2017). The choice between these materials often depends on the balance of biocompatibility, mechanical properties, processability, and specific requirements of tissue engineering application.

### Technologies

Numerous technologies, such as surface modification techniques, nanotechnology, electrospinning technology, and three-dimensional (3D) bioprinting technology, have been applied in the construction of tissue engineering scaffolds. These technologies aim to enhance the physicochemical properties and biocompatibility of the materials, thereby providing a more effective 3D space for cell growth, attachment, proliferation, and differentiation (Cohen et al. 1993).

The composition and structure of the scaffold materials determine their properties, among which the surface characteristics significantly influence cell adhesion, proliferation, and differentiation. Surface modification techniques were used to analyze and optimize the surface properties of scaffolds to enhance cell-material interactions, modifying the material surface in various ways to enhance cell-material interactions (Zhao et al., 2024). For example, by incorporating collagen and coatings with varying concentrations of gelatin methacrylate, surface properties were altered to increase cell adhesion and proliferation (Abraham et al. 2024). The application of nanotechnology in tooth regeneration has been used to manipulate and track SCs survival, migration, and differentiation during regeneration (Bruchez 2011; Ferreira et al. 2008). Alternatively, nanotechnology can deliver genes and proteins into SCs or create artificial microenvironments to induce differentiation (Dalby et al. 2008; Harris et al. 2010). Wei et al. utilized a chemically modified microRNA (miRNA)-loaded tetrahedral-framework nucleic acid nanostructure to enhance DPSCs proliferation and migration and augment their paracrine signaling to endothelial cells, showing great potential for application in pulp regeneration (Wei et al. 2024).

Nanofiber scaffolds fabricated through electrospinning technology mimic the ECM, featuring a high surface area and excellent microstructural properties with highly interconnected porous networks, making them an excellent choice for scaffolds (Bottino et al. 2015; Liu et al. 2012). Wang et al. indicated that nanofibrous scaffolds enhance the adhesion, proliferation, and odontogenic differentiation of hDPSCs more effectively than solid-walled scaffolds (Wang et al. 2011). Moreover, by adjusting the electrospinning process parameters, nanofibers with different diameters, arrangements, and compositions can be prepared, controlling the scaffold’s pore size, porosity, fiber thickness, and internal and external geometries to meet specific tooth regeneration needs (Chen et al. 2018). Zhang et al. utilized electrospinning to fabricate collagen-based 3D fibrous scaffolds with three distinct mean pore sizes (approximately 20, 65, and 145 μm) and compared their effect on pulp regeneration. Among them, the scaffold with a uniform pore size of 65 μm exhibited the highest levels of odontogenic gene expression (*Dspp* and *Dmp1*), protein expression (DMP1), mineralized area ratio, and formation of vascular pulp-like tissue, proposing its potential as an alternative for pulp regeneration (Zhang et al. 2022). In addition, electrospinning can serve as a drug delivery vehicle, achieving controlled and targeted drug delivery (Zhao et al. 2024). Studies have proposed a nanofiber-based intracanal drug delivery system, wherein nanofiber scaffolds impregnated with antibiotics are implanted into infected root canals (Bottino et al. 2013). The controlled release of antibiotics can effectively eliminate canal infections, and after inducing bleeding, they can serve as a matrix for SCs, maintaining the viability of the SCs to promote the repair and regeneration of pulp tissue.

3D bioprinting technology offers new possibilities for custom-made tooth construction. Traditional scaffold material preparation methods face challenges in constructing complex tooth structures with high precision (Vijayavenkataraman et al. 2018). However, 3D bioprinting technology can print bioinks loaded with one or more cells and cytokines into complex 3D biomimetic tissues or organs with certain functions (Kang et al. 2016). By precisely controlling the printing precision, scaffolds with structure similar to that of the natural teeth can be manufactured, and cells and cytokines can be embedded in the scaffolds to further enhance their bioactivity. In hard tissue repair, Sodeyama et al. successfully fabricated a polymer-infiltrated ceramic network composite using 3D printing technology, which exhibited hardness similar to that of enamel and an elastic modulus similar to that of dentin, making it an ideal material for dental restoration (Sodeyama et al. 2021). Chen et al. used adult dogs as a model and found that the mineralization degree of dental pulp SCs transplantation using a 3D-printed hydroxyapatite (HA)/polylactic acid scaffold was significantly higher than that of the acellular scaffold control group (Chen et al. 2021a). As regards pulp regeneration, Han et al. coprinted hDPSC-laden bioink with polycaprolactone to produce dentin-pulp complexes with patient-specific shapes and successfully achieved localized differentiation in vitro (Han et al. 2019). Subsequently, they developed a new demineralized dentin matrix particle-based bioink and successfully fabricated 3D cellular constructs with patient-specific shapes and sizes, significantly enhancing the odontogenic differentiation of DPSCs (Han et al. 2021). Regarding whole-tooth regeneration, Smith et al. and Monteiro et al. successfully constructed 3D GelMA hydrogel tooth bud constructs, which exhibited many of the characteristics of natural tooth buds (Monteiro et al. 2016; Smith et al. 2018). These results demonstrate the potential of using 3D bioprinting technology to produce patient-specific composite tissues. However, the balance between printing precision and bioactivity remains a challenge in this field.

## Advances in clinical trials for tooth regeneration

With the accumulation and maturity of laboratory research, tooth regeneration has entered the clinical trial stage. Clinical trials for tooth regeneration have made significant progress in recent years, focusing mainly on the use of SCs technology to promote functional tooth regeneration, with some clinical trials showing positive results.

In dental pulp regeneration, over 20 relevant clinical trials have been conducted, demonstrating significant potential for application. Xuan et al. has demonstrated that hDPSCs implantation can regenerate 3D pulp tissue equipped with blood vessels and sensory nerves, and it can increase the root length and reduce the width of the apical foramen compared with traditional apexification (Xuan et al. 2018). Nakashima et al. successfully regenerated pulp tissue containing sensory nerves by transplanting DPSCs into the root canals of mature teeth (Nakashima and Tanaka 2024). These trials observed no adverse events, indicating the clinical safety of DPSCs implantation. Studies have combined DPSCs with scaffolds and cytokines for transplantation, which have shown a certain degree of functional recovery of the pulp tissue (Meza et al. 2019; Nakashima and Iohara 2017). Given the relative difficulty in extracting autologous SCs, the recruitment of autologous SCs through cell homing technology has become a more feasible option. Although cell homing technology and the use of biomaterials promote pulp regeneration in mature necrotic teeth to some extent, the operation is complex, and controversy remains over whether regenerative pulp treatment can truly reconstruct the genuine pulp tissue. Therefore, more large-scale studies are needed to provide higher-level evidence (Jiang et al. 2017; Nageh et al. 2018; Shivashankar et al. 2017).

For enamel regeneration, given the developmental mechanisms of enamel, methods to obtain enamel through organ cell culture and cell engineering are still in the laboratory stage. Synthesizing enamel-like apatite materials through physicochemical means can be also performed. Currently, studies have successfully engineered an enamel analog by simulating the hierarchical structure of natural enamel, and its performance has exceeded that of natural enamel and previously manufactured bulk enamel-inspired materials (Zhao et al. 2022). Shao et al. developed a new type of calcium phosphate ion clusters that can establish a biomimetic crystalline-amorphous mineralization frontier to induce the epitaxial growth of tooth enamel (Shao et al. 2019). However, these studies are currently in the in vitro experimental stage and require further assessments of safety and stability. At present, the clinical research of enamel tissue regeneration is mainly achieved through enamel surface remineralization methods, such as the use of fluorides, toothpaste, enamel matrix derivatives (Cao et al. 2014), and synthetic self-assembling peptides (Alkilzy et al. 2018; Brunton et al. 2013).

These studies have indicated that tooth regeneration offers new treatment possibilities for tooth damage and disease. Although the clinical application of tooth regeneration treatment is still in its early stages, the preliminary results are encouraging. Long-term follow-up of patients receiving new treatment methods is still needed to assess the durability of the treatment effect and any potential long-term side effects. Further collection and analysis of clinical data are needed to verify these findings and explore the factors that affect treatment success to continuously improve the treatment methods and improve the therapeutic effect.

## Challenges and prospects

The development of tooth regeneration technology has brought revolutionary progress to oral medicine. However, before it can be widely applied, a series of challenges must be overcome, and future development directions require in-depth consideration.

### Clinical translation challenges

The ultimate goal of tooth regeneration is to achieve whole-tooth regeneration. Although some studies have managed to regenerate whole teeth in situ in animal models, challenges to overcome before this can enter the clinical research phase remain. First, obtaining an appropriate cell source is necessary. At present, most raw materials used to achieve whole tooth regeneration still rely on embryonic SCs, which means the presence of ethical and security concerns in the future (Wu et al. 2019). Adult SCs, while avoiding ethical issues to some extent, have limited pluripotency. The emergence of iPSCs has addressed this issue to some degree, although it also brings other problems. Even if the right cells are found, correctly inducing these cells to gain odontogenic potential remains a challenge. Although current research has produced structures similar to teeth, these structures are often small in size, difficult to control in shape, and differ from the natural tooth morphology (Honda et al., 2005). Moreover, studies have successfully generated structurally correct teeth; however, the process requires further research to enhance its efficiency and controllability (Honda et al. 2007; Nakao et al. 2007). The masticatory capacity of these regenerated teeth, their response to mechanical stress, and perception of nerve stimulation also require further investigations (Ikeda et al. 2009). Moreover, the natural development process of teeth takes a long time; thus, how to accelerate the entire developmental process to achieve rapid tooth regeneration in clinical practice must be considered. Immune rejection is also a challenge. In tooth regeneration, the host’s immune system may attack and reject the transplanted cells or biomaterials, leading to regeneration failure. Therefore, researchers must develop materials with high biocompatibility and explore immunosuppressive strategies to reduce the host’s immune response to transplanted cells.

For tooth regeneration that has entered the clinic stage, such as pulp regeneration, although certain achievements have been made, its technical maturity and controllability limit its widespread application in clinical practice. Standardized procedures for pulp regeneration treatment have not yet been established, including cell acquisition, processing, transplantation techniques, and postoperative management, making practical application difficult. Infection control is particularly crucial. Conversely, the tissue regenerated after pulp regeneration must be stable in the long term to maintain the function and health of the tooth. Currently, the number of clinical studies is limited, and the follow-up time is short, with insufficient data on long-term safety and efficacy. Whether regenerative pulp treatment can reconstruct the real pulp tissue and whether the regenerated pulp will undergo degenerative changes in the long term are unclear, and large-scale clinical trials are needed for further research and validation. In addition, patient selection and indications must be further clarified. Similar to whole-tooth regeneration, pulp regeneration faces issues of cell sourcing and immune rejection. Even if these technologies can be applied to the clinic, raw materials are needed to provide the raw materials for SCs. There is no way to keep the extracted third molars and the replaced deciduous teeth as backup.

In summary, tooth regeneration technologies face challenges such as immune rejection, long-term stability, and ethical and legal issues. In addition, this review focuses on dental tissue, and periodontal tissue regeneration is also an integral part of tooth regeneration. The integration of the regenerated teeth with periodontal support structures to achieve the reconstruction of masticatory function and the perception of mechanical stimulation is also a challenge that cannot be overlooked. Because of the unique systemic characteristics of periodontal tissue regeneration, it is suitable for further exploration in a separate discussion.

### Future directions

Despite the current obstacles, the future trajectory of tooth regeneration technology is promising. Advancements in personalized treatment strategies and interdisciplinary research will provide innovative solutions to existing challenges. Accelerating the clinical translation of research findings is essential to bridge the gap between experimental success and real-world application. This involves conducting more large-scale clinical trials to validate the safety and efficacy of regenerative pulp treatments and other tooth regeneration technologies. Exploring new cell sources and developing methods to reduce immune responses to transplanted cells will enhance the feasibility and success rate of tooth regeneration procedures. Spatial omics and other emerging technologies can bring about major breakthroughs in dental pulp regeneration research. Spatial omics can precisely map cell types and gene expression at the tissue level, helping us gain a deeper understanding of cell–cell interactions and tissue architecture within dental pulp. This paves the way for developing more effective regeneration strategies (Di et al. 2024a, 2024b). This will not only improve the quality of life of the patients but also foster progress in oral medicine.

## Conclusions

Dental regeneration has made remarkable progress in recent years, and the application of tissue engineering technology gradually makes tooth regeneration possible. Dental pulp regeneration has entered clinical trials, and the in situ regeneration of the entire teeth has been successfully achieved in large animals. These achievements have laid the foundation for the clinical application of tooth regeneration treatments in the future, bringing new hope to patients. Despite advancements in tooth regeneration, challenges remain, including how to enhance the quality and functionality of regenerated tissues and ensure the long-term stability of treatments. Future research may focus on the development of novel biomaterials, improvements in the extraction and expansion techniques of SCs, exploration of more effective regenerative strategies, and interdisciplinary research to facilitate further clinical translation.

## Data Availability

Not applicable.

## Abbreviations

SCs: Stem cells
iPSCs: Induced pluripotent stem cells
DPSCs: Dental pulp stem cells
hDPSCs: Human dental pulp stem cells
SHED: Stem cells from human exfoliated deciduous teeth
SCAPs: Stem cells from apical papilla
DFCs: Dental follicle cells
PDL: Periodontal ligament
PDLSCs: Periodontal ligament stem cells
BMSCs: Bone marrow mesenchymal stem cells
ADSCs: Adipose-derived stem cells
GMSCs: Gingival mesenchymal stem cells
BMP: Bone morphogenetic protein
FGF: Fibroblast growth factor
SHH: Sonic hedgehog
DSPP: Dentin sialophosphoprotein
DMP1: Dentin matrix protein 1
Dlx3: Distal-less 3
PTEN: Phosphatase and tensin homolog
KLF5: Kruppel-like factor 5
DE: Dental epithelium
OE: Oral epithelium
IDECL: Immortalized dental epithelial cell lines
CNCLCs: Cranial neural crest-like cells
ECM: Extracellular matrix
dECM: Decellularized extracellular matrix
3D: Three-dimensional
miRNA: MicroRNA
HA: Hydroxyapatite
DPSC-exos: Exosomes derived from DPSCs
PLA: Polylactic acid
PLLA: Poly-L-lactic acid
PCL: Polycaprolactone
PLGA: Polylactic-co-glycolide
GelMA: Gelatin Methacrylate
RA: Ratinoic Acid
